# Geocoding and Social Marketing in Alabama’s Cancer Prevention Programs

**Published:** 2005-10-15

**Authors:** Julianna W Miner, Arica White, Sally Palmer, Anne E Lubenow

**Affiliations:** Alabama Department of Public Health, Social Marketing Branch; Alabama Statewide Cancer Registry, and Sally Palmer, Social Marketing Branch, Alabama Department of Public Health, Montgomery, Ala; Social Marketing Branch, Alabama Department of Public Health, Montgomery, Ala; Office of Communications, National Cancer Institute, Bethesda, Md

## Abstract

The Alabama Department of Public Health (ADPH) is collaborating with the National Cancer Institute to develop detailed profiles of underserved Alabama communities most at risk for cancer. These profiles will be combined with geocoded data to create a pilot project, *Cancer Prevention for Alabama's Underserved Populations: A Focused Approach*. The project's objectives are to provide the ADPH's cancer prevention programs with a more accurate and cost-effective means of planning, implementing, and evaluating its prevention activities in an outcomes-oriented and population-appropriate manner.

The project links geocoded data from the Alabama Statewide Cancer Registry with profiles generated by the National Cancer Institute's cancer profiling system, Consumer Health Profiles. These profiles have been successfully applied to market-focused cancer prevention messages across the United States.

The ADPH and the National Cancer Institute will evaluate the efficacy of using geocoded data and lifestyle segmentation information in strategy development and program implementation. Alabama is the first state in the nation not only to link geocoded cancer registry data with lifestyle segmentation data but also to use the National Cancer Institute's profiles and methodology in combination with actual state data.

## Introduction

The Alabama Department of Public Health (ADPH) estimates that in 2005, more than 24,000 people in Alabama will be diagnosed with cancer, and approximately 10,000 people will die of the disease (1). Health disparities compound this problem and create additional challenges for the public health infrastructure. Cancer incidence and mortality are affected by a wide variety of socioeconomic, behavioral, and other environmental factors, including poverty, race, access to and quality of care, education, obesity, nutrition, and tobacco use, among others (2). The ADPH's cancer prevention efforts are aimed at lowering incidence and mortality for all Alabamians; however, the program's main focus is ameliorating health disparities and reaching underserved populations. In Alabama and across the United States, African Americans bear a higher cancer burden than their white counterparts (3). The socioeconomically disadvantaged are also more likely to have cancer than the general population (4). Reaching poor, rural populations with screening and prevention messages and improving access to treatment and services create additional challenges for public health (5).

The ADPH is working to reduce health disparities by implementing several comprehensive programs offering outreach, education, and cancer screenings to low-income and uninsured populations. Since 1996, the ADPH has provided free breast and cervical cancer screenings to more than 18,000 low-income women (6). Other publicly and privately funded programs, many in partnership with the ADPH, are working concurrently to reach medically underserved communities (7,8).

Eliminating health disparities is one of *Healthy People 2010*'s two overarching goals (9). In addition, the American Cancer Society (ACS) has identified reducing the burden of cancer on the poor and underserved as one of its advocacy priorities (4). However, public health efforts to reduce disparities are challenged by a lack of socioeconomic data that can be linked with data on health behavior and health care use within a relatively small geographic area. In the *American Journal of Epidemiology*, Krieger et al of Harvard's Public Health Disparities Geocoding Project state, "Despite growing recognition of the magnitude and persistence of socioeconomic inequalities in health and the need to address them, few or no socioeconomic data exist in most U.S. public health surveillance databases" (10).

Geocoding technology offers a way to link area-based socioeconomic data and public health surveillance (10). Geocoding is a process of mapping each record in a data set based on a street address and assigning it to a census block group, the smallest geographic unit for which U.S. census data are available. Data from census block groups can be compared with and linked to other data sets. *Healthy People 2010*'s objective 23-3 is to "increase the proportion of all major national, State, and local health data systems that use geocoding to promote nationwide use of geographic information systems (GIS) at all levels" (11). The target is 90% of all public health data systems (11).

Health communications, education, and outreach are increasingly expected to be data-driven and outcomes-oriented (12-14), but these expectations can be difficult to meet for smaller programs, county health departments, or activities targeting rural communities or smaller geographic areas. Simply providing preintervention and postintervention statistics on incidence, screening, and health behaviors can be difficult. Reliable national data sources such as the Behavioral Risk Factor Surveillance System (BRFSS) and the National Health and Nutrition Examination Survey (NHANES) are excellent resources, but when used at the county level (or below) they become less reliable because of small sample sizes (11).

In the actual practice of health promotion, it is not always realistic to expect small or underfunded programs to conduct surveys and focus groups to set baselines for planning and evaluation (15,16). Many state public health agencies function in a limited-resource environment, and often this means prioritizing among many program elements (17,18). In such environments, funds are expected to be allocated for direct services (free screenings, visits with outreach workers or caseworkers, hours of education provided); materials (posters, pamphlets, educational materials); or direct media (radio, television, print and outdoor advertising). Funding sources often limit the amount a program may spend on administrative costs (19,20). In our experience, despite the need to make health promotions more data-driven, limited resources hamper our ability to translate theory into practice.

Alabama's recognition of these problems led to the development of a unique solution, *Cancer Prevention for Alabama's Underserved Populations: A Focused Approach*. This project involves linking geocoded data with other public health databases to plan social marketing activities that will reach communities most at risk for various types of cancer. The ADPH is the first state health department in the United States to license commercial planning and marketing software for this purpose. The ADPH's Bureau of Health Promotion and Chronic Disease began to use this software in 2003. These efforts are being coordinated through the Bureau of Health Promotion and Chronic Disease's Social Marketing Branch.

## Project Goals

*Cancer Prevention for Alabama's Underserved Populations: A Focused Approach *has the primary objective of improving the overall efficacy and cost-effectiveness of cancer prevention messages targeting underserved communities through a pilot project to be conducted from 2004 through 2006. Project goals include 1) the development of profiles of poor and underserved Alabama communities most at risk for various types of cancer; 2) the development of the most effective and cost-efficient ways to reach those communities with prevention messages; 3) the ability to plan, implement, and evaluate cancer prevention activities using valid and reliable data at different geographic levels; and 4) assessment of the value and validity of profiles based on cancer incidence compared with profiles developed from self-reported national health behavior surveys.

## Project Background

### Geocoding Alabama statewide cancer registry data

Gaining access to integrated commercial planning and marketing software was not a quick or an inexpensive process. More than a year was spent working with various programs within the ADPH to discuss the value and usefulness of such an investment. Concerns such as costs, compliance with the Health Insurance Portability and Accountability Act of 1996 (HIPAA), ease of use, and training were addressed. Ultimately, three programs decided to underwrite the cost of the software contract for the first year; during the first year, three additional programs signed on. This arrangement allowed all participating programs to bear a smaller burden of the cost and made the information more widely available. We found that diffusing the cost of the contract across multiple program areas eliminated the cost barrier for most programs that wanted to participate. 

We selected a specific vendor for two primary reasons. First, the vendor had several national public health clients, including the Centers for Disease Control and Prevention (CDC), the Centers for Medicare & Medicaid Services (CMS), the ACS, the National Cancer Institute (NCI), and the National Heart, Lung, and Blood Institute (NHLBI). Both the CDC and the NCI had been sharing this type of data with the ADPH programs for several years, and we saw value in having unlimited, direct access to the data source.

Second, this commercial vendor was the only one to link data from the BRFSS, the U.S. census, and several other national health surveys with its proprietary health care use survey (an annual health behavior survey of 100,000 households) and with lifestyle segmentation clusters. The cluster methodology organizes the U.S. population into 66 segments based on several dozen demographic, geographic, and lifestyle variables as well as consumer-purchase records and media-preference data.

One of the programs to sign on during the first year was the Alabama Statewide Cancer Registry (ASCR). After using the data for several months, the ADPH began the process of geocoding its state cancer registry data in May 2004 with the intention of linking the geocoded cancer data to the various health behavior and socioeconomic databases included in the software, including the lifestyle segmentation clusters. 

Cluster data are linked to a variety of other data within the software, including market research data. These data provide detailed information about each cluster's media preferences (e.g., television shows, newspapers, radio programs), Internet access, and other types of consumer information (e.g., brands of cigarettes smoked, chain restaurants preferred, vehicles purchased). We believed that linking consumer market research, socioeconomic, and health behavior data with 7 years of Alabama state cancer data would offer an unprecedented understanding of who was becoming ill and how best to reach them. 

### The NCI's Consumer Health Profiles

The vendor brought to our attention that one of its clients, the NCI, had developed a series of cluster-based Consumer Health Profiles (CHPs) to help focus cancer prevention outreach to underserved populations. CHPs are designed to profile audiences most in need of cancer education and outreach by potential cancer site (e.g., breast, lung, prostate) based on health behavior and lifestyle information. CHPs incorporate geodemographic, health status, and health care use data to allow the demographic, access-to-care, and behavioral components of prevention and treatment to be better understood. Moreover, CHPs provide lifestyle segmentation data that can be used to design focused outreach to communities based on lifestyle variables such as media preferences, consumer behavior, and the manner in which consumers choose to access information. 

The NCI's profiles have been successfully applied nationally to market-focused cancer prevention and screening messages and have been used extensively at the local and regional levels through the NCI's Cancer Information Service (CIS). For the past 7 years, the CIS Partnership Program staff has used CHPs data to identify underserved and minority populations and to plan and evaluate successful cancer education programs for these groups across the country.

### Collaboration between the ADPH and the NCI

Through the vendor, the ADPH and the NCI decided to collaborate on the project to share expertise and data. We learned that the NCI's profiles were based on self-reported survey data and national data sets. Our data would be specific to the geographic area where implementation would occur and would be based on cancer incidence rates rather than self-reported screening and behavioral data. 

The NCI and the ADPH each identified individuals within their organizations to work on the project. Within the ADPH, representatives from the Social Marketing Branch and the ASCR participated. From the NCI, representatives from various groups within the Office of Communications participated. 

A series of conference calls between project partners over the summer of 2004 resulted in a preliminary program plan and a Memorandum of Understanding that would allow for the free sharing of data between the organizations while preserving confidentiality. 

## Steps to Completion

We have identified the following seven steps to completion of the project:

1. Geocode 7 years (1996–2002) of data from the ASCR and develop a custom software application to allow for various types of data analysis. (This step was completed in November 2004.)
2. Assess the geocoded cancer data to discern trends in incidence and to link incidence data to information on socioeconomic status, access to care, screening behavior, and media or outreach preferences. The analysis will focus on several cancer sites: breast, cervix, colorectal, prostate, lung, and all cancer sites combined. (This step is currently underway.)
3. Link Alabama's findings with the NCI's CHPs for further examination, validation, and strategy development.
4. Collaboratively develop CHPs specific to Alabama for various cancer sites for underserved populations. This project phase will include recommendations on how best to reach profiled communities based on their media or outreach preferences and health behaviors.
5. Select profiles in most urgent need of prevention messages, and conduct additional planning and baseline data collection around the communities where the intervention will be focused.
6. Implement a focused cancer prevention outreach, education, or media campaign.
7. Evaluate and report on the efficacy of the campaign.

## Discussion

We are currently in the process of analyzing the geocoded cancer incidence data and linking the data to information on socioeconomic status, access to care, screening behavior, and media or outreach preferences. Although we are still in the early stages of the project, we have identified several important findings. First, there is a need for ongoing process evaluation. Fortunately, monthly conference calls with all the project partners have served as an excellent way to share suggestions, changes, and ideas on how to improve the project. The commercial vendor has become a partner through this process. This increased involvement has been especially helpful partly because unanticipated alterations to the software application were needed. Because the project has no direct funding and is underwritten by participating programs at the ADPH, the vendor's time, support, and good will have been invaluable.

Second, cancer staging data should be included along with incidence data to ascertain cancer burden. To simplify the process, we did not include staging data in our initial upload of the cancer registry information to the vendor for geocoding. However, such data would have increased opportunities for analysis. For example, by linking this data set with mortality data, we could calculate 5-year survival rates and identify populations with the highest cancer burdens.

Third, there are strengths and weaknesses in using a cluster-based model. Clusters are useful because so much information is already associated with them. However, such clusters are based on national statistics. For example, Alabama's general population is 26% African American, compared with 12.3% of the general U.S. population (21). Therefore, the demographics of several key clusters in our analysis do not match national statistics. It has been necessary to rerun the demographics for each cluster in Alabama at the block-group level to account for these differences. However, we have confidence in the cluster methodology and its applicability to Alabama's population, as does the NCI. Their CHPs, which rely on national data, have been used successfully in regional programs across the country. This Alabama population analysis strengthens the composition and use of the profiles and will help to further validate the project.

Fourth, we need to identify additional uses for data outside of the scope of this project. We are currently working to identify cancer prevention projects in Alabama's Black Belt region that 1) focus on the cancer sites we are assessing, 2) have had interventions occur during 1999 or 2000, and 3) are still in progress. That timeline will allow us to provide these programs with at least 2 years of preintervention and postintervention data to assist them in evaluating their efforts. We hope that this information will assist them with managing their programs and increasing their competitiveness in securing funds.

Providing ongoing projects with this information would be useful for us because it would give us additional experience in applying our methodology and would allow us to examine outcomes data months ahead of what we had anticipated. It would also give us the opportunity to further disseminate this information, thus making the best possible use of our investment in the software and furthering the mission of public health by providing support to grassroots cancer prevention efforts.

Fifth and finally, there is a need to conduct literature searches on cancer incidence, cancer sites, socioeconomic status, geocoding in public health data systems, and a variety of other issues related to the project. We have found that it was useful to place both the process and the preliminary findings in a broader context. This has also yielded the opportunity to speak with public health professionals across the country engaged in similar research who have offered valuable advice and feedback.

The findings of this project are preliminary, and no outcome data are yet available. We hope to have such evaluative information in the next 12 to 18 months. By describing the project and the reasons for its inception, we hope to articulate some of the issues facing public health communications and cancer prevention programs and to outline one of the solutions the ADPH Bureau of Health Promotion and Chronic Disease has adopted to address them. While our solution may not be appropriate for our counterparts in state government across the country, we believe there is value in documenting our experience thus far and hope that it may provide some ideas on how to address the challenging environment in which we all function.
